# Off-Road Autonomous Vehicle Semantic Segmentation and Spatial Overlay Video Assembly

**DOI:** 10.3390/s26061944

**Published:** 2026-03-19

**Authors:** Itai Dror, Omer Aviv, Ofer Hadar

**Affiliations:** School of Electrical and Computer Engineering, Ben-Gurion University of the Negev, Beer Sheva 8410501, Israel; avivom@post.bgu.ac.il (O.A.); hadar@bgu.ac.il (O.H.)

**Keywords:** off-road autonomous vehicles, semantic segmentation, confusion-aware loss, cross-domain generalization, semantic video compression, video encoding, spatial overlay video

## Abstract

Autonomous systems are expanding rapidly, driving a demand for robust perception technologies capable of navigating challenging, unstructured environments. While urban autonomy has made significant progress, off-road environments pose unique challenges, including dynamic terrain and limited communication infrastructure. This research addresses these challenges by introducing a novel three-part solution for off-road autonomous vehicles. First, we present a large-scale off-road dataset curated to capture the visual complexity and variability of unstructured environments, providing a realistic training ground that supports improved model generalization. Second, we propose a Confusion-Aware Loss (CAL) that dynamically penalizes systematic misclassifications based on class-level confusion statistics. When combined with cross-entropy, CAL improves segmentation mean Intersection over Union (mIoU) on the off-road test set from 68.66% to 70.06% and achieves cross-domain gains of up to ~0.49% mIoU on the Cityscapes dataset. Third, leveraging semantic segmentation as an intermediate representation, we introduce a spatial overlay video encoding scheme that preserves high-fidelity RGB information in semantically critical regions while compressing non-essential background regions. Experimental results demonstrate Peak Signal-to-Noise Ratio (PSNR) improvements of up to +5 dB and Video Multi-Method Assessment Fusion (VMAF) gains of up to +40 points under lossy compression, enabling efficient and reliable off-road autonomous operation. This integrated approach provides a robust framework for real-time remote operation in bandwidth-constrained environments.

## 1. Introduction

Autonomous systems are expanding rapidly, driving a demand for robust perception technologies capable of navigating challenging, unstructured environments. While urban autonomy has made significant progress by leveraging standardized features and lane geometry, off-road environments pose unique challenges, including dynamic terrain, irregular object scales, and the absence of established communication infrastructure. The deployment and reliability of these autonomous agents rely heavily on Vehicular Ad-hoc Networks (VANETs) and V2V/V2I communication protocols. However, as the density of autonomous vehicles increases, these communication channels face significant spectrum scarcity and congestion, leading to a marked degradation in Quality of Service (QoS) and increased packet loss [1]. Traditional raw video transmission is often too “expensive” in terms of bandwidth for these congested field environments, where maintaining high throughput is technically prohibitive. To address these bottlenecks, there is an urgent need to shift from traditional bit-level syntactic transmission to task-oriented semantic paradigms. By prioritizing the exchange of “meaning” over raw data, systems can overcome the latency inherent in remote perception. Research indicates that even when cloud-based resources are available, local processing remains the key to keeping latency within operational bounds for real-time navigation [2]. Extracting semantic features at the edge allows the system to prioritize safety-critical information, ensuring that the vehicle remains operational even under constrained network conditions.

This research addresses these challenges by introducing a novel three-part solution for off-road autonomous vehicles:

1. A Novel Off-Road Dataset: We present a large-scale dataset (14,879 images) specifically designed to tackle the variability of unstructured environments. By providing a more realistic and comprehensive training foundation than existing benchmarks, this dataset enables perception models to generalize more effectively to challenging off-road conditions.
2. Confusion-Aware Loss (CAL) Function: To enhance pixel-level classification accuracy, we propose this novel loss component as a complement to standard Cross-Entropy (CE). By leveraging a confusion matrix computed after each epoch, CAL identifies and penalizes systematic classification errors, enabling the model to distinguish visually similar terrain features better. Empirical results using an NVIDIA SegFormer model demonstrate a significant performance gain, with mean Intersection over Union (mIoU) increasing from 68.66% to 70.06%. The generalization of this approach is further validated through consistent performance gains on the Cityscapes benchmark.
3. Spatial Overlay Video Encoding Scheme: Building on our semantic segmentation results, we developed a new spatial overlay video encoding scheme to optimize data transmission. This method significantly reduces bandwidth requirements by intelligently combining a high-fidelity Region of Interest (ROI) from the original video frame with background elements derived from the semantic segmentation output. This approach ensures that crucial details are preserved for a remote operator. At the same time, less critical areas are compressed more efficiently, significantly improving video quality and situational awareness for real-time remote driving.

In this work, we present a holistic approach that integrates a purpose-built dataset, an innovative loss function, and an adaptive video compression scheme. By tightly coupling perception accuracy with communication efficiency, this research underscores the potential of semantic-aware systems to enable reliable autonomous operation in the most demanding real-world applications.

## 2. Background

This section examines the current state of semantic segmentation research as it pertains to autonomous off-road navigation. Unlike urban settings governed by strict lane geometry and standardized features, off-road environments are characterized by unstructured terrain, irregular object scales, and highly dynamic environmental conditions. These complexities necessitate a shift from traditional bit-level syntactic transmission to task-oriented semantic paradigms that prioritize the exchange of “meaning” over raw data to overcome the bandwidth bottlenecks and latency inherent in vehicular perception. We review foundational datasets and evaluate state-of-the-art architectural frameworks that balance real-time processing constraints with high classification accuracy in high-variance natural domains. Furthermore, we situate our work within the emerging field of semantic communication, exploring how importance-aware compression can enhance situational awareness where traditional geometric methods often fail.

### 2.1. Datasets for Off-Road Environments

Standard semantic segmentation research has historically focused on urban and interurban environments, supported by mature benchmarks such as Cityscapes [3] and CamVid [4]. However, the highly structured nature of these datasets, relying on clear lane markings and geometric consistency, makes them poorly suited to the unpredictable features of natural terrain. Off-road autonomous navigation requires models trained on unstructured, high-variance data, yet effective open-source solutions in this domain remain scarce. To fill this gap, our research leverages an extensive, specialized off-road dataset of semantically labeled images to provide a robust perception framework explicitly tailored to the complexities of natural environments.

#### 2.1.1. Foundational Forest and Terrain: Dataset and Model Architecture

The Freiburg Forest dataset [5], published in 2016, represents a fundamental contribution to off-road semantic segmentation. It marked a crucial turning point in the field by shifting the focus from structured urban environments to the complexities of unstructured natural terrain. As a pioneering effort, it provided a benchmark for researchers developing autonomous off-road vehicles. The dataset comprises 15,000 multispectral images at a resolution of 300 × 300 pixels, with 366 pixel-wise ground truth manually annotated. Alongside the dataset, the authors introduced the UpNet architecture, a deep convolutional neural network (DCNN) that significantly outperformed contemporary state-of-the-art models in both qualitative and quantitative metrics. In particular, UpNet was nearly twice as fast as the following best technique, demonstrating a significant leap in computational efficiency. Furthermore, the research highlighted the power of multimodal fusion: the authors found that combining RGB and Enhanced Vegetation Index (EVI) data using a late-fusion convolutional approach yielded the highest segmentation accuracy. This fusion technique demonstrated that leveraging multispectral information is key to accurately interpreting the subtle features of complex forest environments.

#### 2.1.2. Multimodal Sensor Fusion and Off-Road Benchmarks

The authors of [6] present a semantic mapping system for an All-Terrain Vehicle (ATV) that fuses geometric data from a LiDAR sensor with semantic information derived from a custom convolutional neural network (CNN). This system constructs a 2.5D grid map to provide a richer environmental representation, enabling the vehicle to differentiate between true obstacles and traversable features, such as tall grass. To support this, the authors introduced the Yamaha-CMU Off-Road (YCOR) dataset, a publicly available resource comprising 1076 images annotated with eight classes using a polygon-based interface. By integrating this semantic map with a path planner, the system demonstrated successful autonomous navigation across challenging off-road terrains that would be impassable for a purely geometric system.

Off-Road Freespace Detection (ORFD) is a benchmark dataset introduced by [7] for off-road freespace detection in autonomous systems. The dataset comprises 12,198 synchronized pairs of LiDAR point clouds and RGB images, collected under highly diverse environmental conditions to ensure robust model generalization. To effectively harness these multimodal data streams, the authors proposed OFF-Net, a Transformer-based model featuring a novel dynamic cross-attention mechanism that intelligently fuses information from camera and LiDAR sensors in real time.

Experiments conducted on the ORFD dataset demonstrate that OFF-Net significantly outperforms existing models designed initially for on-road environments. These results underscore the critical need for specialized research and tailored tools to achieve reliable off-road autonomy, particularly through attention-based architectures that can handle the complexities of unstructured terrain.

The RELLIS-3D dataset [8] is a multimodal dataset designed for autonomous off-road navigation research, explicitly addressing the limitations of urban-focused benchmarks such as Cityscapes. Collected on the RELLIS Campus of Texas A&M University, it provides a crucial benchmark for semantic segmentation in unstructured environments. The dataset includes synchronized raw sensor data from multiple sources, including LiDAR and RGB cameras. A key contribution of this work is the extensive ground-truth annotations, comprising 13,556 point-wise LiDAR labels and 6235 pixel-wise image annotations across 20 classes. These classes include specific types of off-road terrain, such as mud, puddles, and rubble. The authors’ evaluation of state-of-the-art models revealed significant performance degradation compared to results on urban datasets, highlighting the unique challenges posed by unstructured features and severe class imbalance in off-road domains. To directly address the severe class imbalance and low pixel density of tail-end classes observed in datasets like RELLIS-3D and RUGD, Viswanath et al. [9] proposed the OFFSEG framework. Rather than forcing a single network to resolve all fine-grained textures, OFFSEG employs a two-stage pipeline. First, it pools the highly variant dataset categories into four broad macro-classes: Sky, Traversable, Non-Traversable, and Obstacles, enabling robust, real-time segmentation using the BiSeNetV2 multi-branch architectures. In the second stage, the framework isolates the “Traversable” region. It applies K-Means color clustering alongside a MobileNetV2 classifier to re-extract mission-critical sub-classes, such as mud, gravel, and puddles. While this hierarchical approach effectively mitigates baseline class imbalance, its reliance on a secondary, heuristic color-clustering stage underscores the ongoing need for end-to-end loss formulations that can intrinsically resolve off-road class ambiguity within a single neural network pass. Taking a LiDAR-centric approach to traversability, Shaban et al. [10] similarly formulated off-road navigation as a semantic terrain classification problem based on four distinct cost levels (free, low-cost, medium-cost, and lethal). To address the sparsity of point clouds, the authors introduced the Bird’s Eye View Network (BEVNet), a recurrent neural network that directly predicts a local 2D traversability map from LiDAR inputs. By integrating 3D sparse convolutions with a Convolutional Gated Recurrent Unit (ConvGRU), BEVNet efficiently aggregates geometric and semantic features over time while dynamically correcting odometry noise. Furthermore, the architecture incorporates an inpainting module to predict terrain classes in unseen or occluded spaces, inherently learning to filter out irrelevant overhanging obstacles without relying on rule-based heuristics. Evaluated extensively on the RELLIS-3D dataset, this temporally consistent framework demonstrated superior performance compared to traditional geometric aggregation baselines, further highlighting the necessity of unified spatio-temporal reasoning for robust costmap construction in complex terrains.

#### 2.1.3. Specialized and Extreme-Scale Off-Road Data

TNS, a system detailed in [11], addresses the challenging problem of autonomous navigation for excavators in unstructured construction environments. Its core innovation is a method for generating real-time traversability maps by intelligently fusing geometric features from a LiDAR sensor with semantic data from an RGB camera. To mitigate the scarcity of domain-specific training data, the authors introduced the Complex Worksite Terrain (CWT) dataset, comprising 669 annotated construction site images. Integrating TNS with a modified spatial overlay planning algorithm increased navigation success rates by 49.3% compared to purely geometric approaches. This significant improvement demonstrates the system’s capacity to safely guide autonomous machinery through high-risk obstacles, such as steep drop-offs and rock piles, where traditional geometric methods often prove insufficient.

More recently, the ORAD-3D dataset [12] significantly expanded the scope of off-road benchmarks. As a large-scale multimodal dataset, it provides comprehensive coverage of various terrains and environmental conditions, addressing the limitations of earlier, smaller-scale datasets. It provides a modern foundation for evaluating 3D occupancy and path planning in high-complexity, unstructured scenes.

#### 2.1.4. Data Integration and Synthetic Augmentation

In [13], a novel framework is presented to robustly combine existing off-road datasets into a single cohesive resource, addressing the critical challenges of data variety and annotation inconsistency. The core innovation involves utilizing Ontology Web Language (OWL) to fuse four major datasets systematically, DeepScene’s Freiburg Forest, RELLIS-3D, RUGD, and YCOR, to create a cross-integrable unified resource. This work demonstrates three primary applications: enabling the training of more accurate, general-purpose machine learning models; allowing intelligent data querying based on hierarchical classes; and facilitating the use of automated reasoners to identify inconsistencies in labels and model predictions. Ultimately, this framework provides a methodical approach to standardizing perception data, accelerating the development of algorithms for navigation in complex, unstructured environments.

To address the persistent scarcity of diverse real-world training data, recent research has explored the utility of simulated environments. Małek et al. [14] introduced the OffRoadSynth dataset, demonstrating that pretraining models on high-fidelity synthetic data significantly enhances performance when fine-tuning on limited real-world samples. Furthermore, the authors proposed a systematic framework to mitigate the “fragmented data” problem by fusing multiple existing resources, including Freiburg Forest, RELLIS-3D, and YCOR, into a single, robust, and unified resource. This approach helps overcome the inherent limitations of individual datasets, such as narrow geographic diversity or inconsistent class labeling, providing a more comprehensive foundation for training generalized off-road perception models.

### 2.2. Semantic Segmentation Frameworks

Although robust datasets provide a solid foundation, selecting an appropriate neural network architecture is critical for balancing real-time processing constraints with classification accuracy in off-road autonomous systems. Unstructured environments impose unique demands on feature extraction that differ significantly from urban settings. Consequently, we evaluated several state-of-the-art semantic segmentation frameworks to assess their architectural suitability and robustness when deployed in high-variance natural domains. Expanding on the potential of simulation, Wijayathunga et al. [15] proposed a high-fidelity multimodal synthetic dataset generation framework specifically for unstructured terrain. This 2026 study addresses a critical bottleneck in off-road robotics: the need for perfectly synchronized multimodal data, including RGB, depth, and thermal streams, that are often difficult to align in the field. By leveraging advanced simulation engines, the framework generates diverse environmental conditions and complex terrain geometries that challenge the limits of traditional perception models. Their findings suggest that such high-fidelity synthetic environments are not merely substitutes for real-world data but essential tools for training autonomous robots to handle high-risk edge cases, such as extreme slopes or occluded obstacles, thereby bridging the “reality gap” and ensuring safer deployment in unpredictable natural domains.

#### 2.2.1. Real-Time Multi-Branch Architectures

A notable advancement in real-time segmentation is PIDNet [16], which incorporates principles from classical control theory into its architecture. Specifically, the model is influenced by Proportional Integral Derivative (PID) controllers, with a three-branch structure that balances different levels of feature processing. By adding a “Derivative” branch to focus on boundary recognition, the network aims to mitigate the “overshoot” problem commonly observed in traditional two-branch models, such as DDRNet [17].

Although PIDNet achieves high performance on urban benchmarks, reaching 78.6% mIoU on Cityscapes, our internal experiments using its open-source implementation on off-road data yielded an mIoU of only approximately 50%. This significant performance degradation is likely attributable to the high sensitivity of the boundary detection (Derivative) branch to the ambiguous, stochastic transitions inherent in natural terrain. Unlike structured city streets, off-road environments lack the sharp geometric contrasts and well-defined borders required for the PID-based derivative component to function effectively, leading to increased noise and classification errors at terrain interfaces.

Similarly, BiSeNet (Bilateral Segmentation Network) [18] employs a dual-path architecture, with a spatial path to preserve high-resolution detail and a context path to capture a broad receptive field. Although BiSeNet achieves an impressive balance of speed and accuracy, attaining 68.4% mIoU at 105 Frames Per Second (FPS) on the Cityscapes benchmark, it remains sensitive to category imbalance, which is prevalent in natural environments.

DDRNet (Deep Dual-resolution Network) [17] addresses similar trade-offs by combining a dual-resolution architecture with a Deep Aggregation Pyramid Pooling Module (DAPPM). This module provides multiscale contextual information while minimizing computational overhead by operating on low-resolution feature maps. Despite their efficiency, both BiSeNet and DDRNet often struggle to maintain high accuracy in complex off-road scenes. In these scenarios, the demand for fine-grained feature resolution often conflicts with the high computational requirements of processing unstructured textures, leading to performance trade-offs that often sacrifice precision for speed.

#### 2.2.2. Transformer-Based Architectures

SegFormer [19] represents a paradigm shift in semantic segmentation by integrating a hierarchical Transformer encoder with a lightweight All MLP decoder. Unlike traditional CNN-based models or earlier Transformer architectures such as SETR [20], SegFormer eliminates the need for fixed positional encoding. This design choice makes the network uniquely robust to variations in image resolution between the training and inference phases, a critical advantage in off-road scenarios where camera vibration and environmental factors can frequently affect image scale.

The SegFormer-B5 variant demonstrates remarkable efficiency, achieving 84.2% mIoU on the Cityscapes benchmark while maintaining a significantly higher throughput than SETR. However, its high-capacity architecture remains heavily dependent on the quality of the annotations and the scale of the training dataset. Despite these data requirements, the seamless integration of local and global attention mechanisms allows SegFormer to capture the complex, nonlinear spatial dependencies and fine-grained textures characteristic of unstructured natural environments.

#### 2.2.3. Transfer Learning and Domain Adaptation

Transfer Learning and Domain Adaptation: The scarcity of off-road data often necessitates the use of transfer learning. Research by Sharma et al. [21] suggests that smaller, lightweight versions of architectures like DeconvNet may be more effective for the specific simplicity of certain off-road scenes compared to larger urban-trained models. Their study also highlights the role of synthetic datasets as intermediate domains. In contrast, domain-specific synthetic data can boost performance; overly simplistic simulations may lead to “negative transfer,” where pre-trained knowledge hinders real-world accuracy. By synthesizing these findings, our work adopts the NVIDIA SegFormer backbone, initialized with ImageNet-pretrained weights, to establish a robust baseline for general visual features. We address the specific hurdles of “class ambiguity” and “data scarcity” in unstructured environments using a novel CAL, evaluating its generalizability and performance on the Cityscapes benchmark before fine-tuning the model on our specialized off-road dataset.

### 2.3. Semantic Communication for Teleoperation

#### 2.3.1. Foundations of Semantic Communication in Vehicular Networks

The survey by Ye et al. [22] provides a comprehensive overview of semantic communication in the Internet of Vehicles (IoV), highlighting its ability to address spectrum scarcity and high latency by conveying semantic meaning rather than raw data bits. The authors categorize key technologies into semantic information extraction, communication architectures, and resource management and highlight the use of deep learning models such as Swin Transformers and GANs for efficient feature extraction and scene reconstruction. For autonomous driving perception, the research reviews how multimodal fusion (integrating LiDAR, cameras, and GPS) into a Bird’s-Eye View (BEV) semantic space can maintain high accuracy even in low-SNR environments. Furthermore, the article explores the integration of reinforcement learning for dynamic resource allocation and federated learning for privacy-preserving distributed training while identifying critical challenges in computational overhead, adversarial security, and the need for global standardization.

#### 2.3.2. Task-Oriented Transmission and Edge Perception

The proposed VIS-SemCom system [23] addresses the bandwidth challenges of vehicle-to-everything (V2X) communications by shifting from traditional image transmission to a task-oriented semantic paradigm. By using a Swin Transformer backbone, the framework extracts multiscale semantic features that prioritize safety-critical objects such as vehicles, pedestrians, and obstacles over less relevant background data, such as the sky or fences. This approach employs a custom importance-aware loss function and an Online Hard Example Mining [24] (OHEM) strategy to ensure that even small or rare objects are segmented with high precision, a vital factor for reliable decision-making in autonomous driving. Experimental results demonstrate that this architecture significantly outperforms conventional JPEG and LDPC-based transmission schemes, particularly in challenging wireless environments. The system achieves approximately 70% data reduction while maintaining a 60% mIoU, effectively mitigating the “cliff effect” often observed in traditional digital communications at low signal-to-noise ratios. Furthermore, it provides a coding gain of nearly 6 dB and improves the segmentation accuracy of critical objects by 4%, offering a robust and bandwidth-efficient solution for real-time vehicular perception. The IndiSegNet framework [25] introduces a lightweight architecture specifically designed to handle the chaotic, unstructured environments typical of developing regions. Built on a ResNet50 backbone, the model incorporates two novel components: the Multiscale Contextual Feature (MSCF) module to capture irregular object scales and the Encoded Feature-Refining (EFR) module to enhance boundary detail for thin structures. IndiSegNet achieves state-of-the-art performance on the Indian Driving Dataset (IDD) with a 67.2% mIoU while maintaining an impressive real-time inference speed of 112 FPS on a Jetson Nano. Extensive field testing across diverse terrains, including monsoonal urban areas and foggy mountainous routes, confirmed the model’s robustness, with less than 2.5% variance in mIoU and significantly improved detection for safety-critical classes such as pedestrians and riders.

## 3. Dataset Preview

To facilitate robust autonomous navigation and semi-autonomous teleoperation in unstructured environments, we introduce a novel off-road dataset comprising 14,879 high-resolution images (1280 × 720 pixels). The data was collected across 16 distinct non-urban locations in four countries, capturing a wide variety of dirt roads and complex off-road terrains. Each image is paired with a dense, pixel-wise semantic segmentation mask annotated for 16 specific terrain and object categories.

A critical feature of this dataset is its unbalanced class distribution, which accurately reflects the inherent statistical challenges of real-world operational environments. The dataset is partitioned into training (10,415 images), validation (2233 images), and testing (2231 images) subsets.

**Figure 1:**
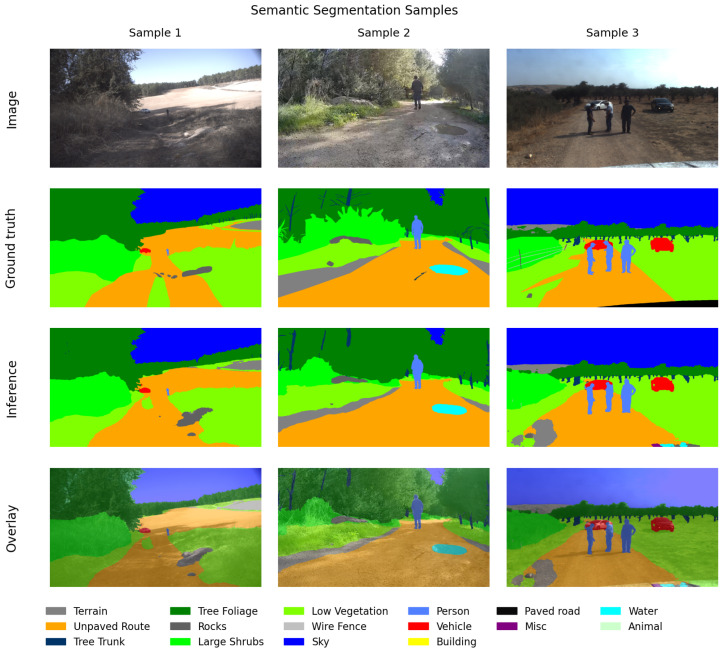
Qualitative evaluation of the proposed semantic segmentation model on the off-road test set. Row 1: input RGB images; Row 2: manual ground-truth annotations; Row 3: model inference results; Row 4: overlay of the inference results over the original manual RGB images.

Figure 1 presents representative samples from the test set to demonstrate data diversity and model performance. The figure is structured for comprehensive qualitative evaluation: Row 1 displays the raw input RGB images; Row 2 shows the manual ground-truth annotations; Row 3 illustrates the inference output generated by our proposed model; and Row 4 presents the spatial overlay, representing the image superposition of the original RGB images and the model inference results for contextual verification.

**Figure 2:**
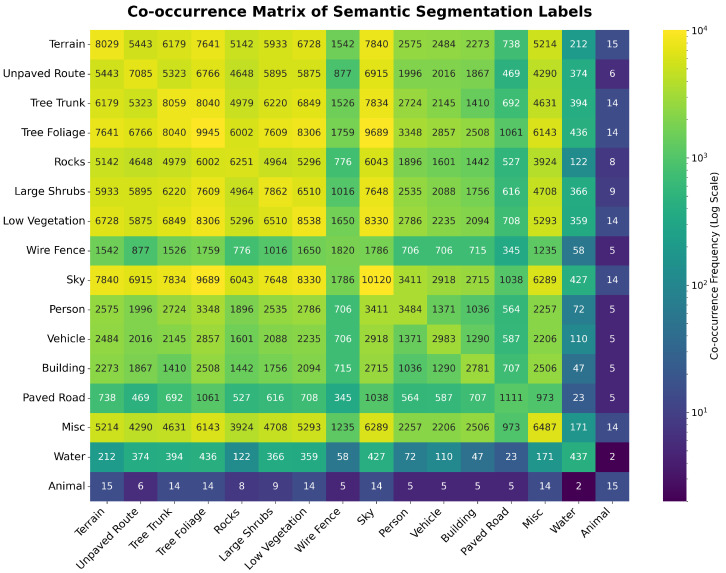
Co-occurrence matrix visualizing the statistical frequency and contextual associations between the 16 semantic classes. The color intensity represents the co-occurrence frequency on a log scale.

The co-occurrence matrix, shown in Figure 2, is a symmetric heat map that effectively visualizes the contextual relationships among the 16 semantic labels in the dataset. In this representation, the axes correspond to the distinct semantic classes. The main diagonal entries quantify the total number of images containing a specific label, providing a direct metric of class frequency. Conversely, the off-diagonal cells represent the number of images where two distinct labels co-occur.

This tool provides critical insight into the statistical associations inherent in the data, distinguishing strong environmental priors such as the frequent co-occurrence of ‘Sky’ and ‘Tree Foliage’ (9689 images) from rare events like the ‘Animal’ and ‘Water’ pairing (2 images). Crucially, this matrix serves as a data exploration tool, distinct from a confusion matrix used for performance evaluation. Its purpose is to reveal the spatial context and logical dependencies of the scenes, which directly inform our sampling strategies and the design of the CAL function.

## 4. Off-Road and On-Road Raw Video Acquisition

To evaluate the proposed model’s performance in real-world scenarios, approximately 100 uncompressed video clips were acquired at two distinct off-road locations near our university campus. The acquisition setup utilized a DSLR (Digital Single-Lens Reflex) camera (Panasonic, Osaka, Japan) equipped with an Olympus M.Zuiko Digital 7–14 mm 1:2.8 lens (Olympus, Tokyo, Japan) operated at a 7 mm focal length. outputting a raw HDMI signal in Full HD resolution (1920 × 1080) at 30 FPS with YUV420p color subsampling. The camera was secured to the hood of an SUV using a high-stability suction mount without a gimbal, thereby capturing the natural vibrations and ego-motion typical of off-road driving. The wide-angle lens provided a Horizontal Field of View (HFOV) of 104.4°, ensuring a comprehensive perspective of the unstructured terrain.

The raw HDMI signal was captured using an HDMI-to-USB converter connected to a mobile workstation. To prevent frame drops and ensure data integrity during high-bitrate acquisition, each 30-s clip was first buffered to system Random Access Memory (RAM) before being committed to Solid-State Storage (SSD). We implemented a temporal monitoring protocol that discarded any clip exceeding its nominal recording duration by more than 2%, indicating potential system-level latency or hardware bottlenecks. To optimize storage efficiency without compromising visual fidelity, the resulting uncompressed clips were archived using the lossless FF Video 1 (FFV1) codec [26] via the FFmpeg framework [27].

**Figure 3:**
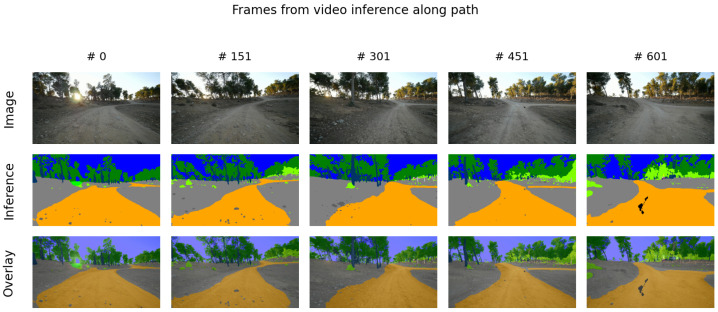
Qualitative evaluation of semantic segmentation performance along an off-road path. The rows display the raw input image (Row 1), the model inference over the original image (Row 2), and the resulting overlay representing the image superposition of the original image and the inference result (Row 3) across a temporal sequence (Frames #0 to #601). The results demonstrate consistent pixel-wise classification in a field-test environment acquired independently from the original dataset.

**Figure 4:**
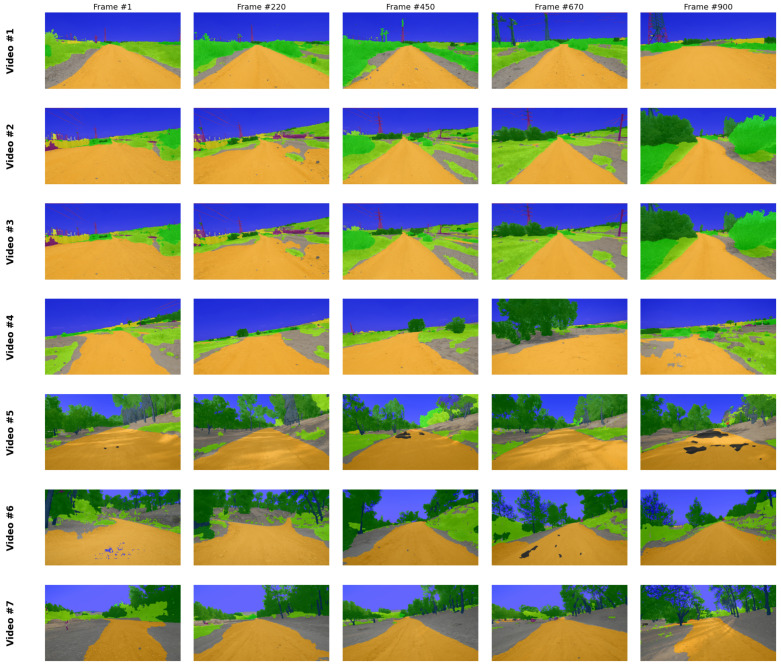
Comprehensive grid of model inference results across seven diverse off-road video sequences. Each row represents a distinct video clip recorded along different segments of the off-road routes across two geographic locations, while columns show the temporal stability of the segmentation from Frame #1 to Frame #900. The consistent identification of the navigable path (orange) and environmental features (e.g., foliage in green, sky in blue) highlights the model’s robust generalization capabilities across varying unstructured terrains.

The trained model’s capacity for real-world deployment and domain generalization is demonstrated through inference on field-test sequences entirely excluded from the training and validation phases. As illustrated in Figure 3, the model maintains high-fidelity pixel-level classification across various off-road environments, effectively managing the dynamic ego-motion and varying lighting conditions inherent in raw sensor data. Further qualitative assessment is provided in Figure 4, which displays a grid of semantic segmentation overlays across seven distinct video sequences. These results underscore the robustness of the system, as it successfully identifies critical features such as the primary navigable path (orange), vegetation (green), and sky (blue) across diverse unstructured geometries not present in the primary dataset.

## 5. Methods

This research focuses on the generation of spatial overlay videos, created by integrating standard video footage with its corresponding semantically segmented output. To generate these segments, we utilized the SegFormer framework [19], implemented through the open-source software provided by Sithu [28].

Semantic segmentation is a critical process in image analysis, enabling the partitioning of an image into distinct, meaningful regions, which is particularly advantageous for target detection and scene understanding in complex off-road environments [19]. SegFormer advances this process by employing a hierarchically structured Transformer encoder paired with a lightweight Multi-Layer Perceptron (MLP) decoder, offering a streamlined yet highly effective approach to semantic segmentation.

A key innovation of the SegFormer architecture is its encoder, which generates multiscale features without requiring positional encoding. By eliminating positional codes, the model avoids performance degradation typically caused by interpolating codes when testing and training resolutions differ. Complementing this is a simplified decoder design that prioritizes efficiency. The MLP decoder aggregates information across multiple layers, successfully combining local and global attention mechanisms to produce robust feature representations.

For our experiments, we employed the MiT-B3 variant of the SegFormer model. The framework offers a range of Mix Transformer (MiT) encoders, notably B0 to B5, which increase in complexity with depth. While the MiT-B5 configuration provides maximum accuracy through its extensive layer count, it incurs significant computational overhead. Conversely, MiT-B0 is optimized for high-speed inference but offers lower precision. We selected MiT-B3 because it offers an optimal trade-off, providing the high precision needed for our analysis while maintaining sufficient computational efficiency for timely processing.

### 5.1. Loss Function

As recent comprehensive reviews emphasize, the strategic design of loss functions is critical for shaping the real-world performance and reliability of deep learning-based segmentation algorithms [29]. Building on this principle, we introduce a new loss component, CAL, which is applied in conjunction with the standard CE loss [30]. Unlike uniform loss functions, CAL specifically penalizes common inter-class errors more heavily than random distributions. These error statistics are derived from a confusion matrix calculated before each training epoch or after a significant number of mini-batch iterations.

#### 5.1.1. Normalized Confusion Matrix

**Figure 5:**
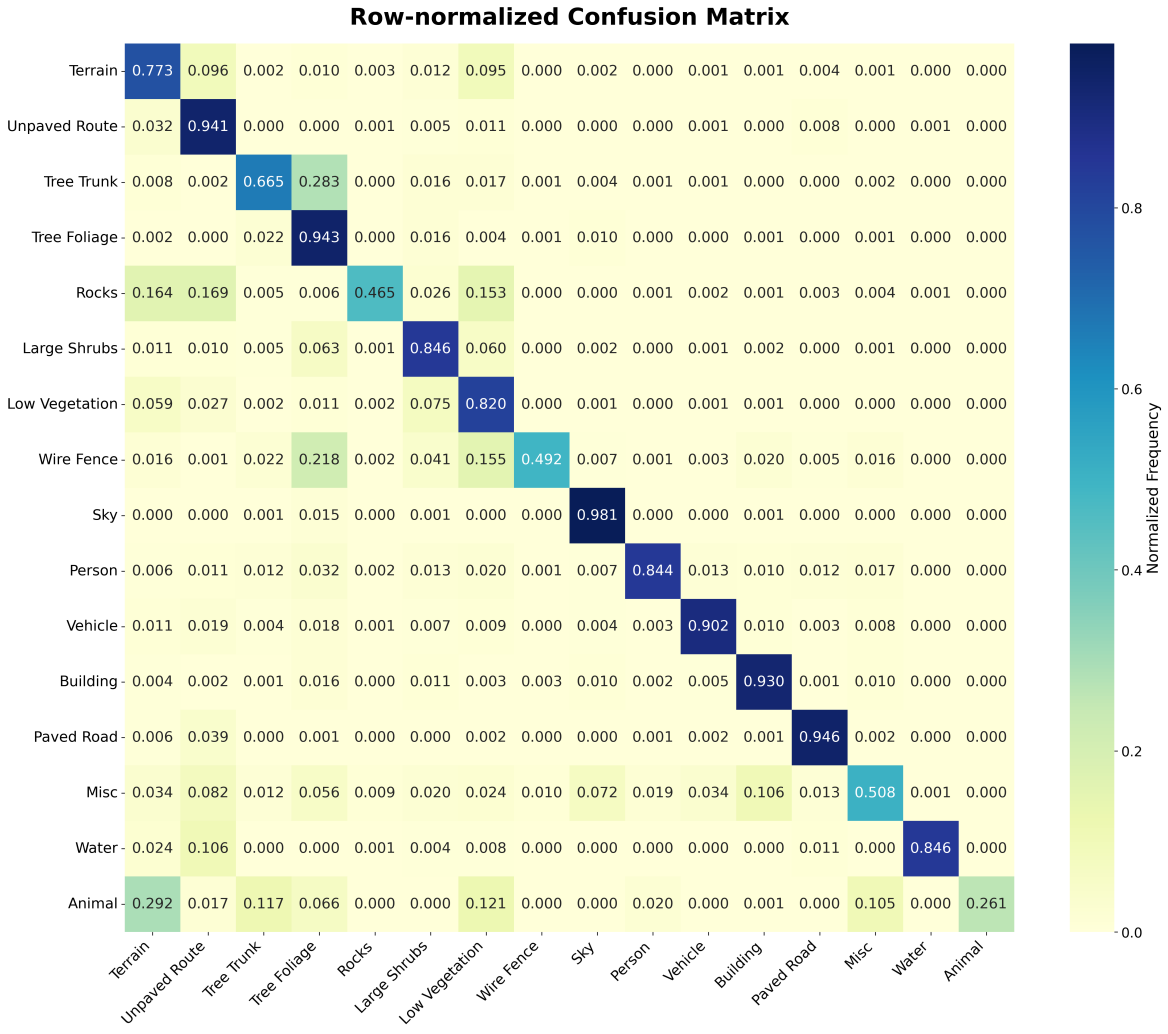
Row normalized confusion matrix.

We utilize a row-normalized confusion matrix, denoted as $CM_{\mathrm{norm}}$, to represent the conditional probability of a model’s prediction given the true class. Each row in the matrix is normalized such that its sum equals 1, as defined by:

(1) $$CM_{\mathrm{norm}}\left[i,j\right]=\frac{CM[i,j]}{\sum_{k=1}^{N}CM\left[i,k\right]}$$

where CM[i,j] denotes the number of instances where true class *i* was predicted as class *j* and *N* represents the total number of classes. This normalization provides insight into the model’s recall by quantifying the percentage of true class instances correctly identified versus those misclassified into specific categories. As illustrated in Figure 5, a model trained solely on CE loss exhibits specific patterns of confusion. For instance, in the “Terrain” row, we observe high false-negative probabilities for “Unpaved Route” (0.096) and “Wire Fence” (0.095), indicating frequent confusion between these visually similar classes. Consequently, a prediction of “Unpaved Route” when the ground truth is “Terrain” will incur a significantly higher penalty than a prediction of an unrelated class like “Building.” Similarly, high-confusion pairs such as Tree Trunk/Tree Foliage (0.283) or Rocks/Vegetation are identified and penalized in proportion to their confusion frequency.

#### 5.1.2. CAL Formulation

The CAL function is designed to weight misclassifications based on the strength of the confusion observed in $CM_{\mathrm{norm}}$. For a single pixel *i*, the loss is calculated as:

(2) $${\mathrm{CAL}}_{i}=-\sum_{j=1}^{N}I\left(j\neq y_{i}\right)\;\cdot \;\log\left(1-p_{i,j}+\epsilon \right)\;\cdot \;CM_{\mathrm{norm}}\left[y_{i},j\right]$$

where:

- $y_{i}$ is the ground truth class label for pixel *i*.
- $p_{i,j}$ is the network’s predicted probability for class *j* at pixel *i*.
- $I(j\neq y_{i})$ is an indicator function that is equal to 1 if class *j* is not the true class $y_{i}$, and 0 otherwise.
- ϵ is a small constant (e.g., 10^−6^) used for numerical stability to prevent the logarithm of zero.

The total CAL for the entire dataset is the mean of the pixel-wise losses across all valid pixels (excluding those with an ignore_label).

(3) $$L_{\mathrm{CAL}}=\frac{1}{|L_{\mathrm{valid}}|}\sum_{i\in L_{\mathrm{valid}}}{\mathrm{CAL}}_{i}$$

where $L_{\mathrm{valid}}$ denotes this set of valid pixels.

The overall objective function is formulated by integrating the proposed CAL with the standard CE loss, defined as follows:

(4) $$L_{\mathrm{CCAL}}=L_{\mathrm{CE}}+L_{\mathrm{CAL}}$$

By incorporating this confusion-aware penalty into the optimization process, the total loss becomes highly sensitive to systematic misclassifications. This penalty explicitly forces the model to better disambiguate between visually or contextually similar classes that are prone to error.

### 5.2. Model Training

The experimental framework was implemented on a single NVIDIA GeForce RTX 4090 GPU with 24 GB of VRAM. The model was optimized using the AdamW optimizer [31] with a weight decay of 0.01 and a batch size of 4. To ensure stable convergence across complex off-road textures, we employed a dynamic learning rate management system. This system utilized a composite scheduling strategy, initiating with a base learning rate of 0.001 and blending polynomial decay [32] with cosine annealing [33] to refine the global minimum search during the final stages of training. This optimization followed a strategic two-phase approach:

- Phase 1 (Baseline): The model was trained for 500 epochs using OHEM loss. To mitigate overfitting and ensure optimal weight convergence, we integrated an early stopping mechanism [34], with the total computation time for this stage spanning 336 GPU hours.
- Phase 2 (Retrain): The model underwent an additional 50 epochs of fine-tuning using the proposed Composite Confusion-Aware Loss (CCAL) function.

**Figure 6:**
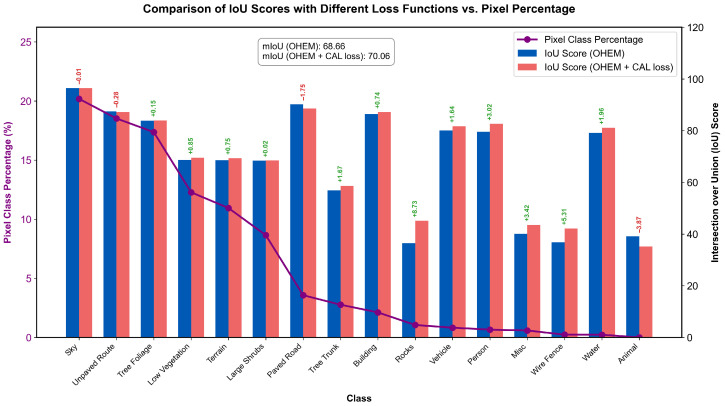
Comparison of per-class Intersection over Union (IoU) scores for two different loss functions: OHEM and the proposed CCAL. The purple line shows the pixel-level percentages for each class, highlighting the severe class imbalance. The mIoU for each loss function is displayed in the top-right inset, demonstrating a performance increase from 68.66% to 70.06% with the integration of CAL.

This sequential training strategy allowed the network to first establish a robust global representation before specializing in disambiguating high-confusion categories. The transition to the CAL-augmented loss successfully improved the mIoU on the off-road test set from 68.66% to 70.06%. As illustrated in Figure 6, the quantitative gains are most pronounced in visually ambiguous and under-represented classes. Our results confirm that the Confusion-Aware Loss specifically targets “tail-end” class errors without compromising the accuracy of dominant environmental features.

**Figure 7:**
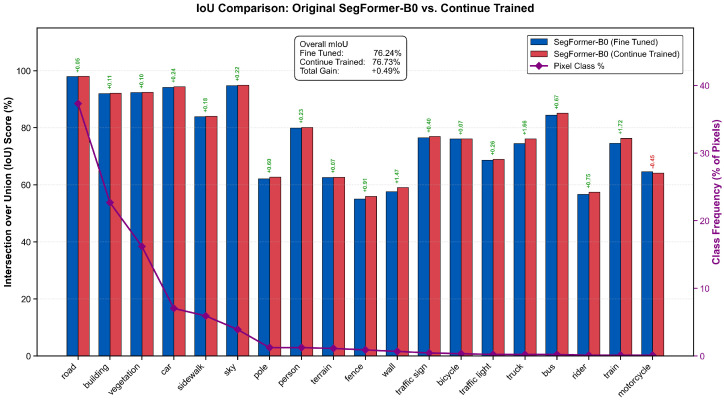
Cross-domain evaluation on the Cityscapes dataset using SegFormer-B0. The plot compares per-class Intersection over Union (IoU) scores between the baseline (CE) and the proposed refinement (CCAL). The purple line represents class frequency, illustrating the performance gains across both dominant and long-tail classes. The top-right inset highlights an overall mIoU improvement from 76.24% to 76.73%, confirming the efficacy of CAL in urban environments.

Figure 7 evaluates the effect of the proposed CAL on cross-domain generalization using the Cityscapes dataset with the SegFormer-B0 model. Table 1 compares the proposed CCAL with several commonly used loss functions for semantic segmentation. To ensure statistical reliability, the results for each evaluated loss function (CCAL, focal, unbalanced CE, Dice, and Tversky) were obtained by retraining the model three independent times using fixed random seeds (10, 42, and 100). As shown in the table, the proposed approach achieves the best performance, reaching a peak mIoU of 76.73%, corresponding to an improvement of 0.49% over the NVIDIA baseline of 76.24% [19]. In comparison, alternative losses such as unbalanced CE and focal loss also improve the baseline, while Dice and Tversky losses yield slightly lower performance in this setting. Nevertheless, the proposed CCAL formulation consistently yields the largest improvement over the baseline. The combined CCAL formulation also demonstrates stable performance across runs, as indicated by the low standard deviation, suggesting that when CAL is applied jointly with CE, it effectively penalizes systematic class confusions that conventional loss functions alone do not adequately address.

Furthermore, Table 2 shows that the performance gain generalizes across multiple SegFormer model variants (B0–B5). Across all architectures, incorporating CAL consistently improves mIoU by up to 0.49% while maintaining the original model complexity since CAL does not introduce additional learnable parameters.

### 5.3. Results of the Spatial Overlaid Video Assembly

**Figure 8:**
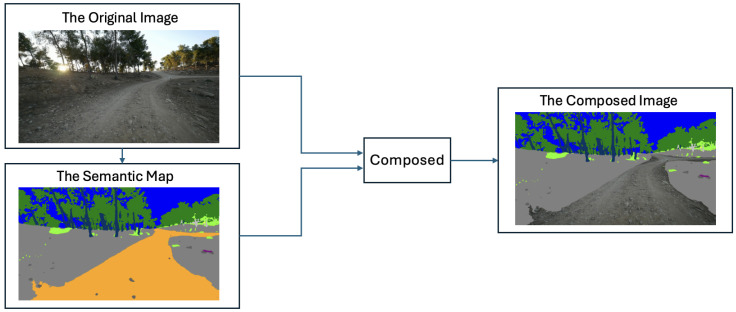
Task-aware spatial overlay video assembly pipeline. The system integrates the original RGB image with its corresponding semantic map to produce a composed frame. This representation preserves high-fidelity visual detail in safety-critical regions (e.g., the navigable path) while abstracting non-essential background areas (e.g., sky and foliage) into semantic color-blocks to optimize data transmission.

The primary goal of this work is to optimize video data for autonomous off-road navigation by reducing visual redundancy without compromising mission-critical information. To achieve this, we developed a novel task-aware video composition method that generates Spatial Overlay frames, as illustrated in Figure 8.

The assembly process begins with a semantic segmentation network that generates a dense, pixel-wise map from an input RGB frame. Rather than treating this map as a standalone output, our approach spatially aligns and overlays it onto the original image. We selectively preserve the most critical regions, such as the navigable off-road path and foreground obstacles, including rocks, low vegetation, and water. In contrast, less essential background classes, such as the sky or distant woods, are replaced with efficiently compressed semantic labels represented by solid colors.

**Figure 9:**
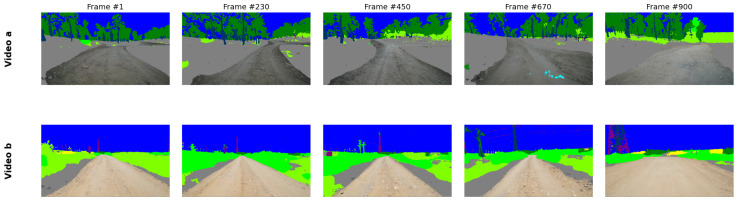
Spatial overlay frames for off-road environments. Representative temporal sequences from two distinct environments: a dense forest (Video a) and an open path with infrastructure (Video b), illustrating the spatial composition output across 900 frames. High-fidelity photographic detail is preserved strictly for safety-critical foreground classes, such as the navigable path. At the same time, non-essential background regions, such as the sky and foliage, are abstracted into semantic color blocks to maximize compression efficiency and situational awareness.

To ensure the integrity of the traversable path, the composed frame is further refined using computer vision techniques, such as connected components analysis, to accurately reintegrate photographic details for key foreground objects located within the ROI. Maintaining photorealism only in safety-critical areas while simplifying non-essential regions, this selective representation strategy significantly improves compression efficiency and reduces data transmission requirements. The resulting performance, which presents our new off-road semantic segmentation model in different video sequences, is presented in Figure 9.

This approach presents a unique challenge to conventional video codecs, which are fundamentally optimized for the statistical properties of natural scenes. Such codecs are often less efficient when processing the hybrid, low-entropy visual patterns characteristic of semantic maps. Nevertheless, our method demonstrated significant gains in lossless compression. Using the FFV1 codec via FFmpeg, the original I420 YUV subsampled video was compressed to 53% of its raw size. In comparison, the newly generated overlaid video achieved even greater compression, reducing the data footprint to 20.2% of the original uncompressed baseline. This method resulted in a lossless reduction to 38% of the overlaid video’s size relative to the compressed original.

While these lossless compression ratios are significantly lower than those achieved with lossy codecs, for instance, shrinking a 1920 × 1080 HD video to 1 Mbps typically requires a compression ratio of approximately 750:1, our method demonstrates considerable potential. It suggests that popular lossy codecs, such as H.264, H.265, H.266, VP9, and AV1 (AOMedia Video 1), could be adapted to compress these spatially overlaid, semantically enriched videos effectively. Such adaptation could lead to more efficient and information-dense video streams, providing a robust representational layer for robotics and autonomous teleoperation.

Table 3 reports the bandwidth requirements for Video a and Video b at an HD resolution of 1920 × 1080 using YUV420p variants under different representation and encoding configurations. The uncompressed YUV420p format serves as a baseline, requiring 746 Mbps for both video sequences. When applying lossless FFV1 compression, the required bandwidth is reduced to 280 Mbps for Video a and 396 Mbps for Video b, corresponding to reductions of approximately 62% and 47%, respectively, compared to the uncompressed baseline.

A substantially larger bandwidth reduction is obtained by employing the proposed segmentation-based semantic representation encoded with FFV1. In this configuration, the bitrates are reduced to 14.5 Mbps for Video a and 24 Mbps for Video b, representing reductions of 98.1% and 96.8%, respectively. Compared to the original uncompressed video, this corresponds to a reduction of approximately 1.5 orders of magnitude, highlighting the strong compression efficiency achieved by replacing dense pixel-domain data with compact semantic representations.

The spatially composite representation exhibits intermediate bandwidth requirements of 117 Mbps for Video a and 151 Mbps for Video b, achieving reductions of approximately 84% and 80%, respectively, compared to the uncompressed baseline. Although this representation requires a higher bitrate than the purely semantic encoding, it still provides a substantial reduction compared to both the raw and lossless-compressed formats.

Across all configurations, consistent trends are observed between Video a and Video b, with absolute bitrate differences reflecting content-dependent spatial and temporal complexity. Overall, these results demonstrate that segmentation-driven semantic representations enable significant bandwidth savings of more than one order of magnitude while maintaining a structured, task-relevant video representation. Although Table 3 reports impressive lossless results, they exceed the direct throughput capabilities typically available for real-time remote data transmission in off-road environments. Accordingly, the remainder of this work focuses on lossy compression schemes that leverage semantic structure to balance extreme compression efficiency with mission-critical visual fidelity.

Table 4 quantitatively evaluates the proposed spatial overlay representation against standard video encoding and purely semantic maps across multiple codecs and bitrates. The results demonstrate that the spatial overlay mode consistently achieves a superior balance between perceptual fidelity and compression efficiency. While the semantic-only mode achieves the highest PSNR and VMAF [35] values due to its simpler visual structure, it lacks photorealistic fidelity in regions that require detailed appearance. In contrast, the spatial overlay approach preserves high-fidelity RGB information in semantically critical ROIs, such as navigable paths and immediate obstacles, while abstracting non-essential background elements. This strategic allocation of bitrate leads to substantial perceptual gains; for instance, at 2 Mbps using the H.264 codec, the spatial overlay achieves a VMAF score of 66.8 compared to 44.0 for standard video. As a result, the spatial overlay representation enables efficient real-time data transmission while maintaining the integrity of mission-critical visual information, validating its suitability for semantic-aware video compression in autonomous navigation scenarios.

Table 5 provides a detailed PSNR breakdown for the AV1 codec, isolating performance across the Overall, ROI, and Background regions. The results demonstrate that the spatial overlay approach effectively preserves ROI quality even under severe bandwidth constraints. For instance, at 1 Mbps, the spatial overlay maintains an ROI PSNR of 31.8 dB, slightly exceeding the 31.7 dB achieved by standard encoding. Notably, the substantial increase in background quality from 30.9 dB in the standard baseline to 34.0 dB in the spatial overlay reveals a significant optimization gap. This surplus in background fidelity suggests that standard codecs could be further modified to improve bit-per-block allocation, potentially maintaining baseline background quality while significantly increasing PSNR and visual clarity in the ROI. While the semantic-only representation yields higher numerical PSNR values due to its abstracted content, it lacks the photorealistic textures essential for human-in-the-loop teleoperation.

Quantitative evaluation across multiple codecs further highlights the perceptual advantages of this task-aware bitrate allocation. As shown in Table 4, the spatial overlay approach consistently improves perceptual quality, achieving VMAF gains of up to +40 points at 2 Mbps. These advantages become more pronounced as bandwidth increases; at 4 Mbps, the method achieves an ROI PSNR of 35.6 dB (a +1.1 dB gain over standard) while simultaneously improving background quality by approximately +5 dB. By successfully prioritizing safety-critical regions as RGB video while abstracting non-essential areas, the proposed scheme supports reliable real-time transmission without degrading the visual integrity required for safe off-road navigation.

### 5.4. System-Level Latency and Teleoperation Impact

As extensively reviewed by Kamtam et al. [36], the end-to-end latency in teleoperation systems fundamentally comprises both local computational delay and network transmission delay. It is critical to note that the proposed CAL is utilized exclusively during the offline training phase. Consequently, while CAL moderately increases the training duration, the architectural complexity of the deployed SegFormer model remains unchanged [19], ensuring it introduces no additional latency or computational overhead during real-time inference. The primary impact on teleoperation response time stems from the spatial-encoding process. In constrained off-road Vehicular Ad Hoc Networks (VANETs), raw video transmission results in severe queuing delays, packet loss, and buffer congestion. By reducing required bandwidth by up to 80% in lossless scenarios and maintaining high visual fidelity at bitrates as low as 1–2 Mbps, the spatial overlay approach effectively mitigates these network bottlenecks. Importantly, the intermediate step of composing these spatial overlay images before video encoding introduces negligible computational overhead; when properly optimized and parallelized on a GPU, this composition runs at high speed, well within real-time constraints. Consequently, this drastic reduction in data footprint directly translates into lower glass-to-glass latency, without being offset by local encoding delays, ensuring that remote operators receive timely, synchronized visual feedback necessary for safe, high-speed teleoperation [37].

### 5.5. Influence on Path Planning and Motion Control

While this study conducts an open-loop evaluation of perception and transmission, the improvements in semantic accuracy carry direct implications for closed-loop Unmanned Ground Vehicle (UGV) motion control. As recent research highlights, complex operational environments can severely degrade raw sensor capabilities in special-purpose Unmanned Ground Vehicles (UGVs) [38]. Therefore, by specifically addressing systematic class confusion, the integration of CAL improves our model’s mIoU from 68.66% to 70.06%, with the most significant gains observed in disambiguating visually similar terrain classes. In the context of autonomous path planning, even marginal improvements in distinguishing the “navigable path” from adjacent “obstacles” drastically reduce noise in the generated 2.5D costmaps or occupancy grids, thereby compensating for environmental sensor degradation and ensuring safer vehicle navigation.

## 6. Conclusions

This research introduces a novel, integrated framework that advances the state of the art in off-road autonomous systems by jointly addressing perception robustness and communication efficiency, two aspects typically treated independently in prior work. Unlike existing approaches that focus either on improving semantic segmentation accuracy or on optimizing video compression in isolation, the proposed method tightly couples these components through a segmentation-centric representation.

Regarding data contributions, prior off-road datasets are limited in scale, geographic diversity, or semantic richness. In contrast, the proposed dataset comprises 14,879 high-resolution images, annotated across 16 semantic classes and collected from 16 locations in four countries, capturing both environmental diversity and the severe class imbalance inherent in real-world operational environments. This scale and variability enable more reliable training and evaluation of segmentation models under realistic, unstructured off-road conditions.

At the algorithmic level, most existing loss functions for semantic segmentation treat all misclassifications uniformly, ignoring systematic confusion patterns between visually similar classes. The proposed CAL explicitly addresses this limitation by dynamically incorporating class-level confusion statistics updated at each training epoch. When combined with cross-entropy, CAL improved segmentation performance on the off-road test set from 68.66% to 70.06% mIoU. Furthermore, it yielded cross-domain gains of up to 0.49% mIoU on the Cityscapes dataset, achieved without any target-domain fine-tuning. These results highlight a key novelty of CAL: enhancing domain-agnostic class separability rather than overfitting to dataset-specific characteristics.

Beyond perception, this work departs from prior semantic video approaches by demonstrating that semantic segmentation can function as an intermediate representation for task-aware video compression. Unlike standard codecs, which are optimized for natural image statistics, the proposed spatial overlay video representation selectively preserves high-fidelity RGB content only in semantically critical ROIs.

Quantitative evaluation shows that, under lossy compression, the spatial overlay approach improves reconstruction quality by up to 5 dB in background regions while maintaining ROI PSNR comparable to that of standard video encoding. Crucially, this surplus in background fidelity reveals a significant optimization gap for future codec development; by adapting bit-allocation strategies to transfer this excess bandwidth from the simplified background back to the ROI, the visual clarity of safety-critical areas could be enhanced even further. At the same time, perceptual quality metrics improve substantially, with VMAF gains of up to +40 points at low and medium bitrates, demonstrating a favorable trade-off between visual fidelity and bandwidth efficiency. Overall, this work introduces a significant practical and theoretical shift from previous research: semantic segmentation is leveraged not only as a perception output but also as a unifying representation layer that jointly improves learning robustness, cross-domain generalization, and communication efficiency [39]. This segmentation-driven paradigm provides a scalable, deployable solution for real-time off-road autonomy and remote driving, enabling safer, more bandwidth-efficient operation in complex, unstructured environments.

## 7. Limitations

While this work presents a robust, holistic solution for off-road autonomous perception, several constraints inherent in the current implementation warrant consideration for future research.

First, although the novel dataset is extensive in scale, it retains an inherently unbalanced class distribution, a pervasive challenge in real-world operational data that can bias model performance toward dominant environmental features. Additionally, while the data was collected across sixteen distinct locations, these remain geographically localized. This potentially constrains the model’s generalizability to the vast array of diverse off-road environments and biomes encountered globally. Furthermore, the robustness of the proposed perception framework relies heavily on the quality of the input data. As highlighted in recent studies, adverse environmental conditions can severely impact raw sensor performance, altering the visual data before the segmentation model processes it [38]. The physical limitations of camera and LiDAR technologies under severe weather such as heavy rain, fog, dust, or low-light night-time conditions often lead to significant degradation in environmental perception [40]. Consequently, future research must evaluate the stability of the proposed spatial overlay encoding under such extreme domain shifts and explore multimodal sensor fusion to mitigate single-sensor failure modes.

Finally, while effective, the proposed spatial overlay video encoding scheme is constrained by the inherent limitations of conventional video codecs. These standards are primarily optimized for the statistical properties of natural scenes and remain less efficient when processing the simplified, low-entropy visual patterns characteristic of semantic maps. This identifies a clear technical opportunity to develop novel compression algorithms specifically tailored to spatially overlaid video streams. Future research should also explore translating this task-aware representation into a broader range of robotic and remote sensing domains beyond off-road autonomous navigation.

## Figures and Tables

**Table 1:** Comparison of the proposed CCAL objective with common loss functions widely adopted for semantic segmentation. Tests utilize the SegFormer-B0 architecture on the Cityscapes dataset against an NVIDIA baseline mIoU of 76.24% [19]. The highest values are highlighted in red, and the second-highest in blue.

Loss Functions	Best Loss mIoU (%)	Mean	Std	Best Improvement (%)
CCAL	76.73	76.49	0.026	0.49
Focal	76.64	76.37	0.061	0.40
balanced CE	76.48	76.27	0.310	0.24
Dice	76.05	75.71	0.033	−0.19
Tversky	75.48	75.18	0.108	−0.76

**Table 2:** Relative performance improvement on the Cityscapes dataset for SegFormer variants utilizing the proposed composite loss. Because CAL does not introduce additional learnable parameters, the model sizes remain identical to their respective baselines. The reported improvement corresponds to the absolute gain in mIoU.

Model Variant	Baseline [19] mIoU (%)	CCAL mIoU (%)	mIoU Improvement (%)
SegFormer-B0	76.24	76.73	0.49
SegFormer-B1	78.55	78.71	0.16
SegFormer-B2	80.83	81.11	0.28
SegFormer-B3	81.53	81.96	0.43
SegFormer-B4	82.33	82.66	0.33
SegFormer-B5	82.26	82.60	0.34

**Table 3:** Lossless bandwidth requirements (Mbps) for Video a and Video b using YUV420p variants across different encoding configurations.

Configuration (1920 × 1080)	Video a (Mbps)	Video b (Mbps)
Original YUV420p baseline	746	746
Lossless FFV1 (Standard)	280	396
Purely Semantic (FFV1)	14.5	24.0
Spatially composite (Overlay), FFV1	117	151

**Table 4:** Lossy quality metrics (PSNR and VMAF) vs. bandwidth for H.264, H.265, and AV1 codecs under three distinct representation modes.

Bandwidth (Mbps)	Codec	Standard Video			Semantic Map			Spatial Overlay	
		PSNR (dB)	VMAF		PSNR	VMAF		PSNR	VMAF
1	H.264	32.0	25.0		33.4	38.1		32.1	26.1
2	H.264	33.8	44.0		41.1	89.1		35.5	66.8
4	H.264	35.7	64.0		43.6	94.8		38.7	79.3
1	H.265	26.1	25.6		30.3	68.7		33.5	51.8
2	H.265	27.1	39.2		32.8	83.3		36.0	70.8
4	H.265	28.4	54.5		35.9	91.5		38.9	86.2
1	AV1	31.2	42.8		35.7	71.7		33.2	54.2
2	AV1	32.5	56.5		38.0	83.1		35.3	71.4
4	AV1	34.2	70.7		41.7	93.3		37.7	85.2

**Table 5:** AV1 PSNR breakdown (Overall/ROI/Background) for three methods at different bandwidths.

Bandwidth (Mbps)	Encoding Scheme	PSNR		
		Overall	ROI	Background
1	Standard	31.2	31.7	30.9
	Semantic	35.7	40.2	34.5
	Spatial Overlay	33.2	31.8	34.0
2	Standard	32.5	32.9	32.3
	Semantic	38.0	42.2	36.8
	Spatial Overlay	35.3	33.5	36.4
4	Standard	34.2	34.5	34.1
	Semantic	41.7	45.5	40.6
	Spatial Overlay	37.7	35.6	39.0

## Data Availability

The CAL retrain, and Spatial Overlay Video Assembly code is available in https://github.com/springaav/Off-Road-Autonomous-Vehicles-Semantic-Segmentation-and-Spatial-Overlay-Video-Assembly (accessed on 10 March 2026).

## Abbreviations

The following abbreviations are used in this manuscript:

ATV	All-Terrain Vehicle
AV1	AOMedia Video 1
AVC	Advanced Video Coding
BEV	Bird’s Eye View
BiSeNet	Bilateral Segmentation Network
CAL	Confusion-Aware Loss
CCAL	Composite Confusion-Aware Loss
CE	Cross-Entropy
CNN	Convolutional Neural Network
CWT	Complex Worksite Terrain
DAPPM	Deep Aggregation Pyramid Pooling Module
DCNN	Deep Convolutional Neural Network
DDRNet	Deep Dual-resolution Network
DSLR	Digital Single-Lens Reflex
EVI	Enhanced Vegetation Index
FFV1	FF Video 1
FPS	Frames Per Second
GAN	Generative Adversarial Network
G-MSC	Generative AI-enhanced Multimodal Semantic Communication
HDMI	High-Definition Multimedia Interface
HEVC	High Efficiency Video Coding
HFOV	Horizontal Field of View
IoV	Internet of Vehicles
LiDAR	Light Detection and Ranging
mIoU	mean Intersection over Union
MiT	Mix Transformer
MLP	Multi-Layer Perceptron
OHEM	Online Hard Example Mining
ORFD	Off-Road Freespace Detection
OWL	Ontology Web Language
PID	Proportional Integral Derivative
PSNR	Peak Signal-to-Noise Ratio
QoS	Quality of Service
QP	Quantization Parameter
RGB	Red, Green, Blue
ROI	Region of Interest
SA-PSNR	Semantic-Aware Signal-to-Noise Ratio
SA-SSIM	Semantic-Aware Structural Similarity
SAC	Semantic-Aware Video Compression
SETR	Segmentation Transformer
SNR	Signal-to-Noise Ratio
SSD	Solid-State Drive
UGV	Unmanned Ground Vehicle
V2I	Vehicle-to-Infrastructure
V2V	Vehicle-to-Vehicle
VANET	Vehicular Ad-hoc Network
VMAF	Video Multi-Method Assessment Fusion
YCOR	Yamaha-CMU Off-Road
YUV	Luminance (Y) and Chrominance (UV) Color Space
